# Activation of PKCβII by PMA Facilitates Enhanced Epithelial Wound Repair through Increased Cell Spreading and Migration

**DOI:** 10.1371/journal.pone.0055775

**Published:** 2013-02-11

**Authors:** Ronen Sumagin, Alex Z. Robin, Asma Nusrat, Charles A. Parkos

**Affiliations:** Epithelial Pathobiology and Mucosal Inflammation Research Unit, Department of Pathology and Laboratory Medicine, Emory University School of Medicine, Atlanta, Georgia, United States of America; University of Cambridge, United Kingdom

## Abstract

Rapid repair of epithelial wounds is essential for intestinal homeostasis, and involves cell proliferation and migration, which in turn are mediated by multiple cellular signaling events including PKC activation. PKC isoforms have been implicated in regulating cell proliferation and migration, however, the role of PKCs in intestinal epithelial cell (IEC) wound healing is still not completely understood. In the current work we used phorbol 12-myristate 13-acetate (PMA), a well recognized agonist of classical and non-conventional PKC subfamilies to investigate the effect of PKC activation on IEC wound healing. We found that PMA treatment of wounded IEC monolayers resulted in 5.8±0.7-fold increase in wound closure after 24 hours. The PMA effect was specifically mediated by PKCβII, as its inhibition significantly diminished the PMA-induced increase in wound closure. Furthermore, we show that the PKCβII-mediated increase in IEC wound closure after PMA stimulation was mediated by increased cell spreading/cell migration but not proliferation. Cell migration was mediated by PKCβII dependent actin cytoskeleton reorganization, enhanced formation of lamellipodial extrusions at the leading edge and increased activation of the focal adhesion protein, paxillin. These findings support a role for PKCβII in IEC wound repair and further demonstrate the ability of epithelial cells to migrate as a sheet thereby efficiently covering denuded surfaces to recover the intestinal epithelial barrier.

## Introduction

Intestinal epithelial cells (IECs) form an important barrier that separates luminal contents from underlying tissue compartments. Epithelial wounds secondary to inflammation and ischemia rapidly reseal to re-establish this critical barrier [1]. Epithelial wound healing is typically mediated by cell migration and proliferation [2]. However, small wounds reseal efficiently by cell migration alone, and this process is referred to as wound restitution. [3], [4]. Migrating IECs undergo morphological changes that involve transitioning from tall columnar to flattened cells that cover denuded surfaces [5]. Spreading epithelial cells polarize to reorient their microtubule organizing center (MTOC) and Golgi apparatus in the direction of the wound [6], as well as extend cellular membrane protrusions at the leading edge referred to as lamellipodia which play an important role in mediating forward cell movement [7], [8]. Additionally, cell movement requires dynamic turnover of focal cell matrix associations and restructuring of the actin cytoskeleton [9].

Protein kinase C (PKC) family members have been implicated in controlling cell migration and proliferation [10], [11], [12]. These proteins mediate signal transduction events that regulate actin cytoskeleton [13] and membrane dynamics [14]. PKCs can be classified into three major subgroups including classical/conventional (α, βI, βII, γ), Ca^2+^ and diacylglycerol-dependent, non-conventional or novel (δ, ε, η, θ, μ), diacylglycerol-dependent, but Ca^2+^ independent, and atypical (ζ, λ), independent of both Ca^2+^ and diacylglycerol. In its unstimulated state, most PKC isoforms reside in the cytosol. However, binding of ligands, such as hormones or growth factors to their membrane receptors results in release of intracellular Ca^2+^ that binds the cytosolic PKC. This results in PKC translocation to the membrane, where it associates with diacylglycerol (DAG) and is transformed into an active enzyme [15]. PKC isoforms are abundantly expressed in IECs [16]. However, given the structural differences among PKC isoforms, and the variability in their subcellular localization, tissue distribution, and substrate specificity, they are known to mediate diverse and often opposing cellular functions [17], [18].

The specific effects of PKC activation on epithelial cell spreading and migration have been investigated in the context of epithelial to mesenchymal transition and tumor carcinogenesis [19], [20], [21]. To date the role of PKCs in intestinal epithelial wound healing is still not completely understood. However, some members of the classical PKC family have been previously suggested to play a role in epithelial tissue repair. For example, PKCγ activation in response to treatment with insulin-like growth factor promoted wound closure [22]. Additionally, PKCβII expression is increased as epithelial cells mature during their migration along the crypt-luminal axis [23].

Phorbol esters, such as phorbol 12-myristate 13-acetate (PMA) can substitute for DAG in stimulating classical and non-conventional PKC isoforms [24]. Thus they are widely used to study the effects of PKC subfamily members on cellular function [25], [26], [27], [28]. In the current work we used PMA activation of PKCs and a panel of broad PKC inhibitors, specific subfamily inhibitors, as well as PKC isoform-specific peptide inhibitors to elucidate the role for PKC activation on IEC wound healing. We found that PMA treatment enhanced IEC wound closure, and that this effect was dependent specifically on PKCβII activation. Furthermore, we determined that increased cell spreading and migration, but not increased cell proliferation primarily contributed to the PMA induced, PKCβII-dependent enhanced wound healing.

## Methods

### Cell Culture

Human intestinal epithelial cells T84s [29] and Caco2 [30] were grown in Dulbecco’s modified Eagle’s medium (DMEM)-F12 50:50 and DMEM respectively, supplemented with 10% fetal calf serum (FCS), 1% L-glutamine, 1% antibiotics, 1% non-essential amino acids, and 1.5% HEPES buffer as previously described.

### Reagents

DMEM, L-glutamine, penicillin/streptomycin, and non-essential amino-acids for cell growth media were obtained from Cellgro (Manassas, VA), FCS was obtained from Atlanta Biologicals (Atlanta, GA). Phorbol 12-myristate 13-acetate (PMA), pan PKC inhibitors Cheleretrine, Calphostin C and Go6976, as well as proliferation inhibitor L-mimosine were obtained from Sigma Aldrich (Houston, TX). PKC isoform-specific peptide inhibitors for PKCβI (KIBI31-1), PKCβII (KIBII31-1), PKCγ (KIG31-1), and a pan classical PKC inhibitor (KIC1-1) were kindly provided by KAI Pharmaceuticals (San Francisco, CA). The development and functions of these PKC isoform-specific peptide inhibitors have been described previously [31], [32], [33]. Additional specific PKCβII inhibitor [34] hydrochloride [1H-pyrrole-2,5-dione,3-(1-methyl-1H-indol-3-yl)-4-[1-[-1(2-pyridinylmethyl)-4-piperidinyl]-1H-indol-3-yl (Enzastaurin, LY-317615) was obtained from LC laboratories (Woburn, MA).

Antibodies, anti-PKCβI (C-16), anti-PKCβII (C-18), and anti-PKCγ (D-4) were purchased from Santa Cruz (Santa Cruz, CA), anti-Na+/K+ pump (C464.6) from Millipore (Billerica, MA), Golgi marker anti-GM130 (35/GM130) and anti-paxillin (177) from BD Biosciences (San Jose, Ca), anti-Phospho-Paxillin (Tyr118, #2541) from Cell Signaling (Boston, MA), and anti-falloidin Alexa Fluor 488/555 from Invitrogen (Grand Island, NY). All secondary antibodies conjugated to HRP were purchased from Jackson ImmunoResearch (West Grove, PA).

### Scratch-wound Assay

IEC monolayers were grown to confluence in 24-well tissue culture plates. A linear mechanical scratch wound was generated in each well using a 20-µl plastic pipette tip attached to low suction [4]. Wounded monolayers were washed once with PBS to remove detached cells and debris, and incubated in medium containing the appropriate treatment. All PKC inhibitors were added 60 minutes prior to the addition of PMA. The rate of cell migration into scratch wounds was measured by determining the surface area devoid of epithelial cells immediately after wounding (t = 0) and at subsequent time points as indicated. The data are shown as percent area at each time point as indicated out of total area at t = 0. For western blot analysis, confluent cell monolayers, grown in 6-well tissue culture plates were scraped 8 times horizontally and vertically to enrich for migrating cells. All experiments were carried out in triplicate, with at least an N = 3.

### Cell Adhesion Assay

Tissue culture 96 well plates were coated overnight with 10 µg/mL of Matrigel™ (356234, BD). Single cell suspensions were generated using a non-enzymatic cell dissociation buffer (Gibco). IECs (150000 cells/condition) were resuspended in 0.1 ml Hanks solution containing Mg^2+^, Ca^2+^ and 0.1% BSA, labeled with BCECF-AM (Invitrogen) and allowed to adhere to a matrix coated/BSA blocked plates for 1 hour at 37°C. When PKC inhibitors were used, IECs were incubated with the specific inhibitor for 60 minutes prior to the adhesion assay. Fluorescence intensity of cells that remained attached to the matrix after 3 consecutive washes was measured using FluoStar Galaxy plate reader at excitation/emission wavelengths of 485/535 nm.

### Immunoblotting

Western blot analyses were performed as previously described [5]. Cells were washed once with HBSS and resuspended in RIPA lysis buffer (150 mM NaCl, 1% NP-40, 0.5% deoxycholic acid, 0.1% SDS, 50 mM Tris, pH 8.0) containing protease and phosphatase inhibitors (Sigma-Aldrich, St Louis, MO). Cell lysates were freeze/thawed in liquid nitrogen, sonicated and cleared by centrifugation. Samples were reduced and boiled, and equal amounts of protein were separated by SDS-polyacrylamide gel electrophoresis and transferred onto pre-activated (methanol) poly (vinylidene fluoride) membranes. Membranes were blocked for 1 h in 3% (wt/vol) BSA in Tris-buffered saline containing 0.1% Tween-20, and incubated with primary antibodies (in blocking buffer, 1 h, RT), followed by secondary antibodies conjugated to HRP, and detected with HyGlo chemiluminescent substrate (Denville Scientific, South Plainfield, NJ).

### Membrane and Cytosol Fractionation

To assay for total protein in membrane and cytosol fractions we used a cell fractionation method as previously described [35]. Briefly, cells were washed with and suspended in Low Salt Buffer (25 mM KCl, 5 mM MgCl, 10 mM Tris-HCl pH 7.8), containing protease and phosphatase inhibitors (Sigma-Aldrich, St Louis, MO) and collected by scraping. Cell samples were lysed by transfer trough 25 g Needle syringe, and centrifuged at 1000 g to remove nuclei and cellular debris. Cytosolic fractions (supernatant) were separated from the membrane fractions (pellet) by centrifugation (15 min, 16000 g). Pellets were resuspended in Low Salt Buffer in volumes equivalent to the supernatant fractions.

### Proliferation

To asses cell proliferation cell cycle analyses and 5-ethyl-2`-deoxyuridine (EdU) incorporation assays were performed. For cell cycle analyses cells were fixed in 70% ethanol (1 hr, 4°C), treated with RNaseA (50 µg/ml, 1 hr, 37°C), and stained with propidium iodide (25 µg/ml, 30 min, 37°C). Samples were analyzed using FACSCalibur flow cytometer (BD Biosciences), and distribution of cell-cycle phases was determined using FlowJo software analysis. For EdU incorporation assay a Click-iT EdU Alexa 488 cell proliferation kit (Invitrogen) was used according to the manufacturer's instructions.

### Immunofluorescense

IECs grown on 13-mm collagen-coated coverslips were fixed and permeabilized with 95% ethanol at −20°C for 15 minutes, blocked with 3% BSA (wt/vol) for 1 hr and incubated with appropriate primary Ab (10 µg/ml) followed by fluorescently labeled secondary Abs, 1 hr at 4°C. Nuclei were stained with ToPro-3 iodide (Invitrogen) and coverslips were mounted on slides using Prolong Gold (Invitrogen). All images were acquired on a LSM 510 confocal microscope (Carl Zeiss, Thornwood, NY) with Plan-Neofluor 60× and 40× objectives. For the assessment of cell directional orientation cells were stained for Golgi and nuclei, and all Golgi oriented within 90 degree angle facing the wound edge were considered correctly oriented.

### Cell Spreading Assay

To assay for cell spreading IECs were seeded on 13-mm collagen-coated coverslips at ∼40 confluency, and allowed to adhere over night. Cells were next stimulated with PMA for 4 hours in the presence or absence of PKCβII inhibitors (KIBII31-1 [36], [37] or Enzastaurin [34]), and cell area before and after treatment was measured and compared to non-activated cells. Both inhibitors yielded similar results. Data are presented as percent cell area out of total area of the field of view. For cell height and individual cell surface area cells were stained for E-Cadherin (a junctional marker) to outline the cell borders. Cell height was quantified from images obtained using confocal microscopy and serial image acquisition in Z-direction. Individual cell surface area was quantified from images obtained using confocal microscopy, and positioning the focal plane at the apical IEC surface. All measurements were performed using ImageJ software.

### Statistics

Statistical significance was assessed by a Student t-test or by one way ANOVA with a Newman-Keuls Multiple Comparison Test using Graphpad Prism (V4.0). Statistical significance was set at P<0.05.

## Results

### PKC Activation with PMA Enhances Epithelial Wound Healing

**Figure 1:**
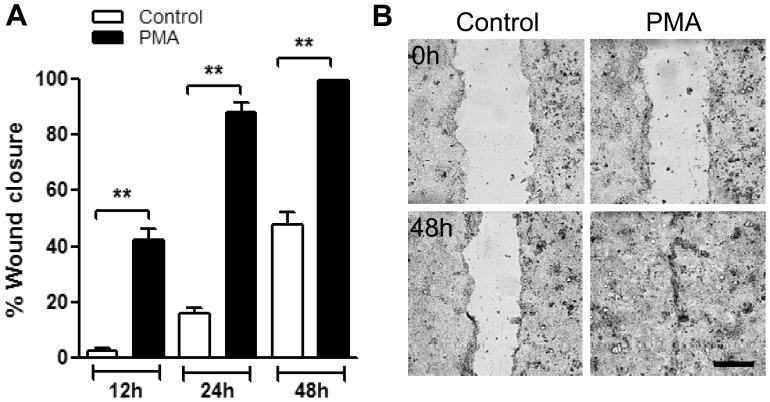
PKC activation with PMA enhances epithelial wound healing. (A) Confluent T84 IEC monolayers were wounded by introduction of a single linear scratch wound. Wound closure was measured over 48 h in unstimulated (control) and PMA activated IEC monolayers as detailed in the methods section. (B) Representative images of unstimulated (control, left panels) and PMA activated (PMA, 200 nM right panels) IEC monolayers immediately after wounding (0 h, upper panels) and after 48 hours (48 h, bottom panels). PMA treatment induced a dramatic increase in wound closure as early as 12 and 24 hours. The bar is 50 µm. N = 4 independent experiments. **significantly different from each other (p<0.01).

PMA activation of PKC isoforms has been shown to enhance epithelial cell growth, proliferation, as well as motility of myeloid cells and fibroblasts [28], [38]. Since epithelial wound healing requires cell migration and proliferation, we hypothesized that PKC activation promotes IEC wound closure. To test this possibility, confluent T84 cells were mechanically wounded (see Methods section) and healing was assessed in the presence or absence of PKC activator, PMA. We observed that in the presence of PMA (200 nM) T84 cell wound healing was dramatically enhanced, 15.9±2.6 fold after 12 hours and 5.8±0.7 after 24 hours (Fig. 1A). Consequently, 24 hours after wounding PMA treated monolayers have recovered 88.0±2.5% of the wounded area compared to 16.2±1.6% in non-treated monolayers. After 48 hours all wounds in PMA treated monolayers were healed, however only 48.2±4.5% of the wounded area recovered in non-treated monolayers (Fig. 1A, and representative images, Fig. 1B). The effect of PMA on wound healing was also confirmed in a different intestinal epithelial cell line, Caco2 (Figure S1). These findings clearly support a role of PKCs in epithelial wound healing.

### PKCβII Mediates PMA Induced Increase in Wound Closure

**Figure 2:**
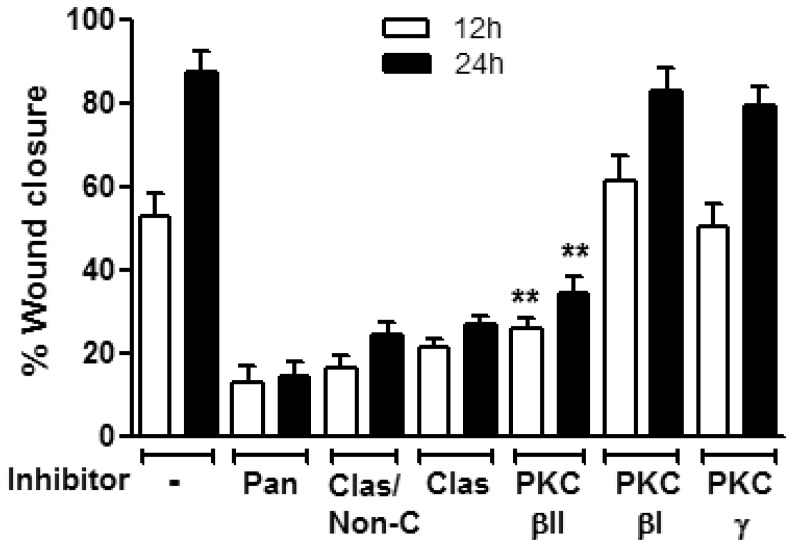
PKCβII mediates PMA induced increase in wound closure. Confluent IEC monolayers were wounded by introduction of a single linear scratch wound, preincubated with the specified PKC inhibitors for 1 hour (Cheleretrine, pan PKC inhibitor (5 µM, pan); Calphostin C, Classical/non-conventional PKC inhibitor (10 µM, *Clas/Non-C*); KIC1-1, classical PKC inhibitor (5 µM, Clas); KIBI31-1, PKCβI inhibitor (1 µM, PKCβI); KIBII31-1 (5 µM, PKCβII); KIG31-1, PKCγ inhibitor (5 µM, PKCβγ) and stimulated with PMA (200nM). The area of the wound was measured at 12 and 24 hours. Inhibition of PKCβII, but not inhibition of other members of the classical PKCs significantly diminished PMA enhanced wound closure. N = 3 independent experiments. **significantly different from control (PMA alone, p<0.01).

PMA activates both classical and non-conventional PKC subfamilies [39]. Thus to examine which PKC subfamily member promotes PMA mediated wound closure we used a panel of broad PKC inhibitors including, the pan PKC inhibitor (Cheleretrine, 5 µM [40]), the inhibitor of classical/non-conventional PKC isoforms, Calphostin C (10 µM) [41] and pan classical PKC inhibitors KIC1-1(5 µM) [36] and Go6976 (0.5 µM) [42]. Since the major effect of PMA on IECs wound closure was observed within the first 24 hours (Fig. 1A) we determined the effect of specific PKC inhibitors at 12 h and 24 h post injury. As shown in Figure 2 pan PKC inhibition as well as inhibition of classical and non-conventional PKCs reversed the PMA-induced increase in T84 IECs wound closure. Importantly, inhibition of classical PKCs alone was sufficient to inhibit the PMA-induced increase in T84 cell wound closure (Fig. 2), confirming that member/s of the classical PKC subfamily are primarily responsible for the PMA effect. Next we used PKC isoform-specific peptide inhibitors for selected classical PKCs including PKCβI (KIBI31-1, 1 µM [37]), PKCβII, (KIBII31-1, 5 µM) and PKCγ (KIG31-1, 5 µM) to identify the specific PKC isoforms that mediate the PMA effect on IEC wound closure. PKCγ and PKCβI inhibition had no significant effect on PMA induced wound closure. However, in contrast, PKCβII inhibition abolished PMA effects on wound closure (Fig. 2), suggesting a role for PKCβII in IEC wound closure in response to PMA activation. To confirm that the inhibition was specific to PKCβII we used an additional PKCβII specific inhibitor Enzastaurin (10 nM, [34]) and observed similar effects (data not shown). These findings were again corroborated in an additional IEC line, Caco2 (Figure S2). PMA treatment has been also shown to induce time dependent depletion of PKC isoforms [43]. Consistent with these findings we found that expression of PKCβII 16 hours after PMA treatment was significantly reduced (Figure S3A). This observation suggests that the reduced effect of PMA on wound repair at 24 hours compared to 12 hours (5.8±0.7 vs.15.9±2.6-fold increase at 24 h vs 12 h, respectively), was due to downregulation of PKCβII expression. Indeed, a decreased PMA-dependent effect on wound repair was evident at 16 hours following PMA treatment (8.3±1.2-fold increase, (Figure S3B).

### PKCβII Translocates to the Cell Membrane Upon PMA Treatment

**Figure 3:**
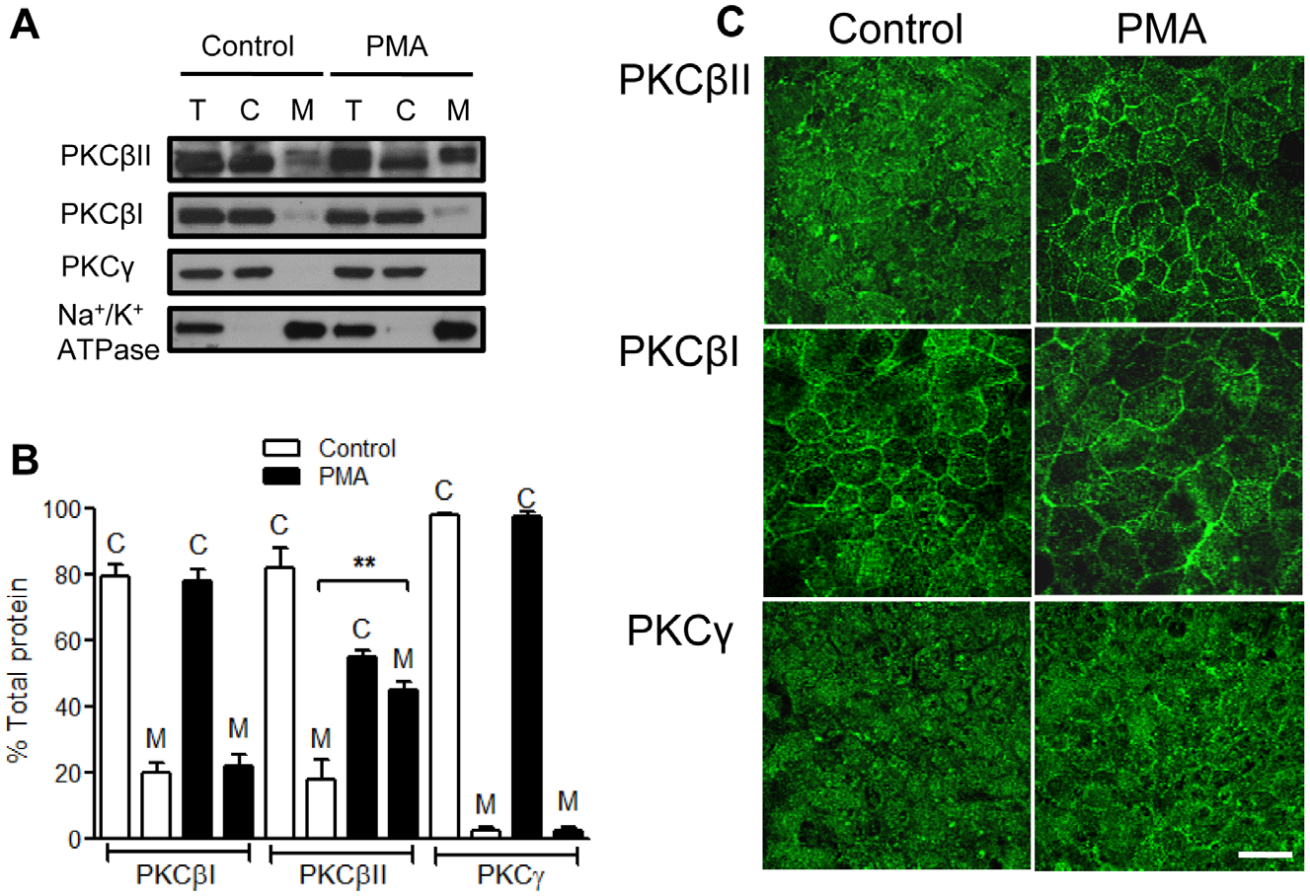
PKCβII translocates to the cell membrane upon PMA treatment. Cellular localization of selected classical PKCs was determined by immnoblotting (**A**) and densitometric analysis (**B**) of the total (T), the cytosolic (C), and the membrane (M) fractions in unstimulated (control) and PMA activated (PMA, 200 nM, 4 h) IECs. **significantly different (p<0.01). N = 4 independent experiments. Following PMA treatment PKCβII translocated to the cell membrane, indicative of its activation. (**C**) PKC isoform localization was also confirmed by immunofluorescence labeling and confocal microscopy. The images show translocation of PKCβII from the cytosol (control) to cell membrane (PMA treatment), and are representative of 3 independent experiments. The bar is 20 µm.

A hallmark of PKC activation is its translocation to the membrane where it interacts with DAG and becomes functionally active [15]. Since we determined that PMA enhanced wound closure was primarily mediated by PKCβII, we next examined the cellular localization of PKCβII, as well as other classical PKCs, PKCβI and PKCγ in T84 IECs stimulated with PMA for 4 hours. As evident from western blot analysis of cytosolic and membrane fractions of T84 IECs (Fig. 3A,B), and confirmed by immunofluorescence labeling and confocal microscopy (Fig. 3C) PKCβII under basal conditions was found to mostly reside in the cytosol, however upon PMA treatment PKCβII translocation to cell membrane was observed (18.1±4.2% at the membrane, control vs. 45.1±2.4% at the membrane, PMA, Fig. 3B), indicative of its activation. PKCβI was found both in the cytosolic and to a lesser extent the membrane fractions and its distribution was not changed after PMA treatment. Similarly, the localization of PKCγ in the cytosolic fraction under basal conditions was not changed with PMA treatment (Fig. 3). Furthermore, we confirmed that PKCβII translocation to cell membrane occurs in leading edge cells after PMA treatment, but not in non-treated wounds (Figure S3C).

### Enhanced T84 Cell Wound Closure Following PMA Activation is not due to Increased Cell Proliferation

**Figure 4:**
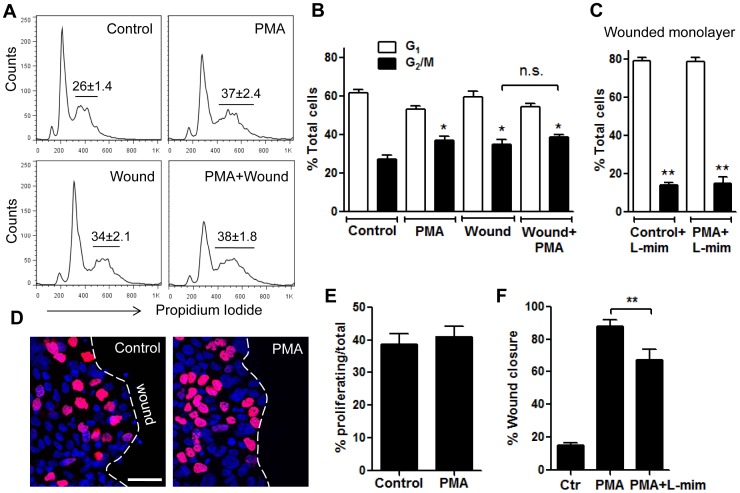
Enhanced T84 cell wound closure following PMA activation was not due to increased cell proliferation. Cell proliferation in non-wounded versus wounded IEC monlayers, unstimulated or after PMA activation (200 nM, 4 h) was assayed using cell cycle analysis (**A,B**) and Edu incorporation (**D,E**). The distribution of cells in G_1_ and G_2_/M phases under indicated conditions is demonstrated by the representative flow diagrams (**A**) and summarized in (**B**). PMA treatment significantly increased the number of cells in G_2_/M phase in non-wounded IEC monolayers, however, there was no significant effect on proliferation of migrating IECs. *significantly different from control (p<0.05). n.s, not significant. N = 4 independent experiments. (**C**) In separate experiments wounded cell monolayers were pretreated with L-mimosine (100 µM, 12 h) stimulated with PMA, and analyzed for cell cycle distribution as described above and compared to unstimulated (control) cells. L-mimosine treatment significantly diminished cell proliferation in scratch wounded cell monolayers, both with and without PMA activation. **significantly different from no treatment (p<0.01). (**D–E**) Cell proliferation at the wound edge was assessed by Edu incorporation. Scratch wounded or confluent cell monolayers were treated with PMA for 4 hr at 37°C before addition of EdU. At least 5 random fields per each condition were analyzed; the data are presented as % proliferating (nuclei stained in purple, EdU)/of total cells (nuclei stained in blue, topro-3) in the field. As shown in representative images (**D**) and quantified in (**E**) PKC activation with PMA had no effect on cell proliferation at the edge of the wound. N = 3 independent experiments. The bar is 50 µm. (**F**) Scratch wounded cell monolayers were either pretreated with L-mimosine (100 µM, 12 h) prior to PMA activation or activated with PMA alone, and wound closure was quantified after 24 hours and compared to unstimulated (control) cells. While L-mimosine treatment significantly decreased wound closure, it accounted only for a small portion of the PMA effect. **significantly different from each other (p<0.01).

Since cell proliferation contributes to IEC wound repair, and PMA has been shown to enhance cell proliferation, we next examined whether PMA enhanced IEC wound healing was due to increased IEC proliferation. To assess the effect of PMA on cell proliferation we used Propidium Iodide staining and flow cytometry of scratch wounded T84 monolayers in the presence or absence of PMA. Consistent with the finding in other cultured epithelial cells [44], PMA treatment of confluent T84 monolayers (non-migrating IECs) significantly increased the number of cells in G2/M phase (from 26.2±1.4%, control to 37.1±2.4%, PMA, Fig. 4A,B) indicative of increased cell proliferation. Interestingly, wounding itself enhanced cell proliferation (34.6±2.1%, Fig. 4A,B), analogous to that observed with PMA treatment. Importantly, PMA stimulation of wounded monolayers failed to further potentiate the increase in cell proliferation, suggesting that PMA has no further effect on proliferation of migrating IECs. These findings were also confirmed by quantifying Edu incorporation into actively proliferating epithelial cells. The number of proliferating cells at the sites of closing wounds (wound edge) was not significantly different between untreated and PMA stimulated wounded monolayers (Fig. 4E and representative images, Fig. 4D). Together these findings suggest that while PKCs might play a role in epithelial cell growth and survival, PMA effects on wound healing was not due to increased cell proliferation. To further confirm that IEC proliferation does not play a major role in PMA-induced increase in wound closure we used L-mimosine, which, has been previously shown to inhibit cells at a regulatory step in late G1 phase, thus preventing cell proliferation [45]. Indeed inhibition of cell proliferation using L-mimosine (L-mim) accounted only for a small fraction (∼20%) of the ∼70% total increase in wound closure after 24 hours PMA activation (Fig. 4F). L-mimosine treatment significantly diminished cell proliferation in resealing wounds, both with and without PMA activation (Fig. 4C) confirming an effect of this inhibitor on cell proliferation.

### PKCβII Promotes lamellipodial Extrusions in Migrating IECs Following PMA Stimulation

**Figure 5:**
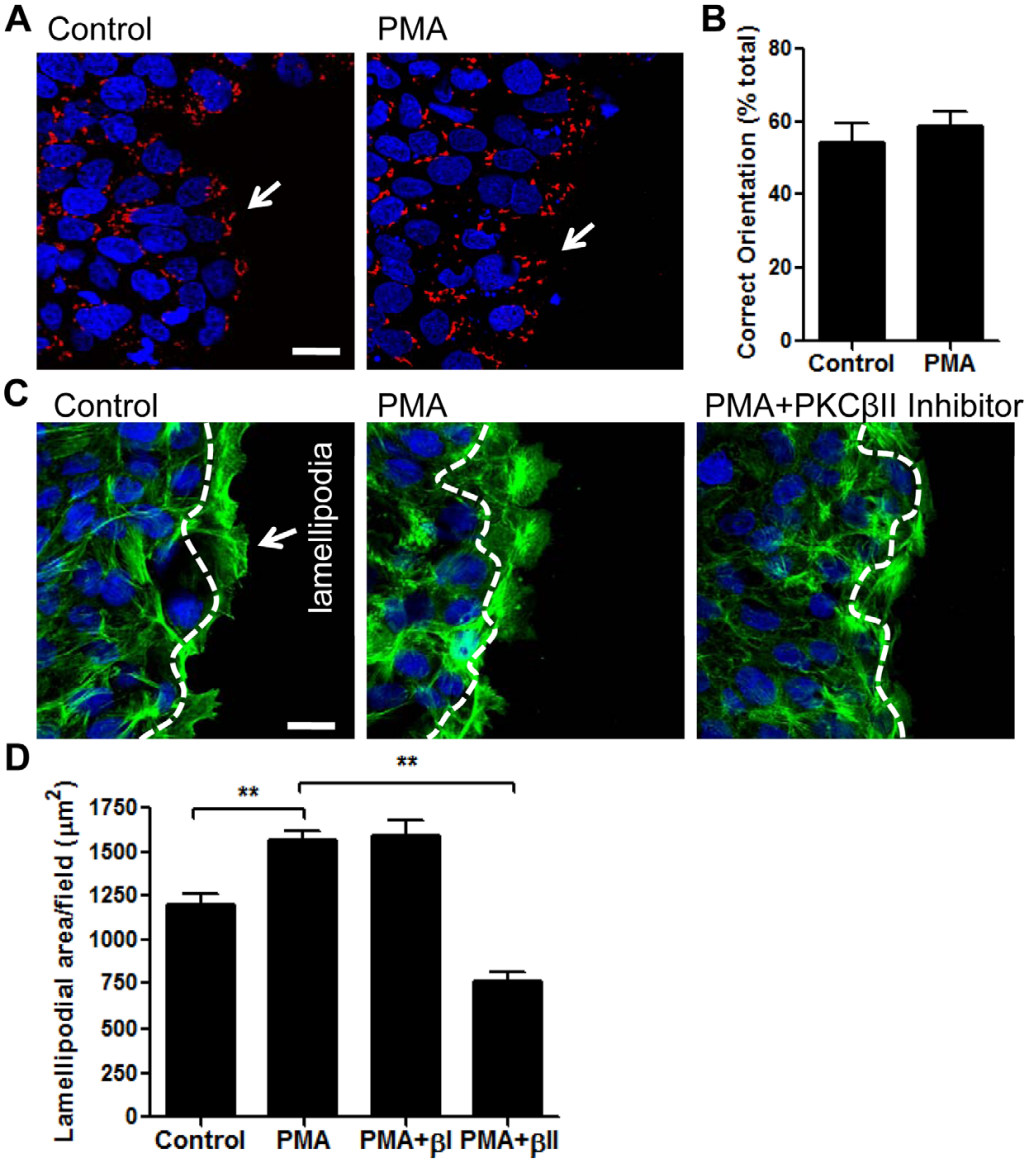
PKCβII mediates extension of cell membrane protrusions in response to PMA stimulation. Scratch wounded cell monolayers were allowed to migrate for 4 hours in the absence (control) pr presence of PMA (PMA). (**A**) Representative images demonstrating the orientation of the Golgi apparatus in migrating cells at the wound edge with or without PMA activation. IECs were then fixed and stained for Golgi (red) and nuclei (blue). The bar is 20 µm. (**B**) Cell orientation in at least 8 randomly selected fields of N = 4 independent experiments were quantified as described in methods. No significant difference was observed in unstimulated versus PMA activated wounded monolayers. White arrows show correctly oriented migrated IECs. The bar is 20 µm. (**C**) IECs were fixed and stained for F-actin (green) and nuclei (blue). Representative images depict extended lamellipodium formation after PMA treatment, which was prevented by inhibition of PKCβII. (**D**) The area of the lamellipodium was defined as area past the nuclei of leading edge cells, as indicated by the white dashed line and quantified following the indicated treatment. At least 5 random fields per each condition were analyzed. N = 4 independent experiments. **significantly different (p<0.01).

Polarization of the leading edge, orientation of the Golgi apparatus in the direction of migration, and lamellipodial extrusions at the leading edge are required for forward epithelial cell movement [7]. To determine IEC polarization in the direction of migration, we first examined the localization of Golgi apparatus in control and PMA activated migrating T84 IECs. We did not observe significant differences in the directional polarity of the leading edge in PMA stimulated cells vs. control migrating IECs (Fig. 5A,B). Next we examined lamellipodium formation in control and PMA activated migrating T84 IECs. Prominent lamellipodia were observed as early as 4 hours after wounding in both control and PMA activated migrating cells. However, PKC activation by PMA resulted in significantly larger lamellipodia at the leading edge compared to those observed in control migrating cells (area between the nuclei and the cell edge as indicated by the white dashed line, 1565±56.8 vs 1197.2±65.6 µm^2^, respectively, Fig. 5D, and representative images Fig. 5C). Inhibition of PKCβII but not PKCβI significantly attenuated lamellipodia formation, supporting a role of these PKC isoforms in mediating cellular protrusions at leading edge.

### PKCβII Activation by PMA Enhances Assembly of Focal Adhesions

**Figure 6:**
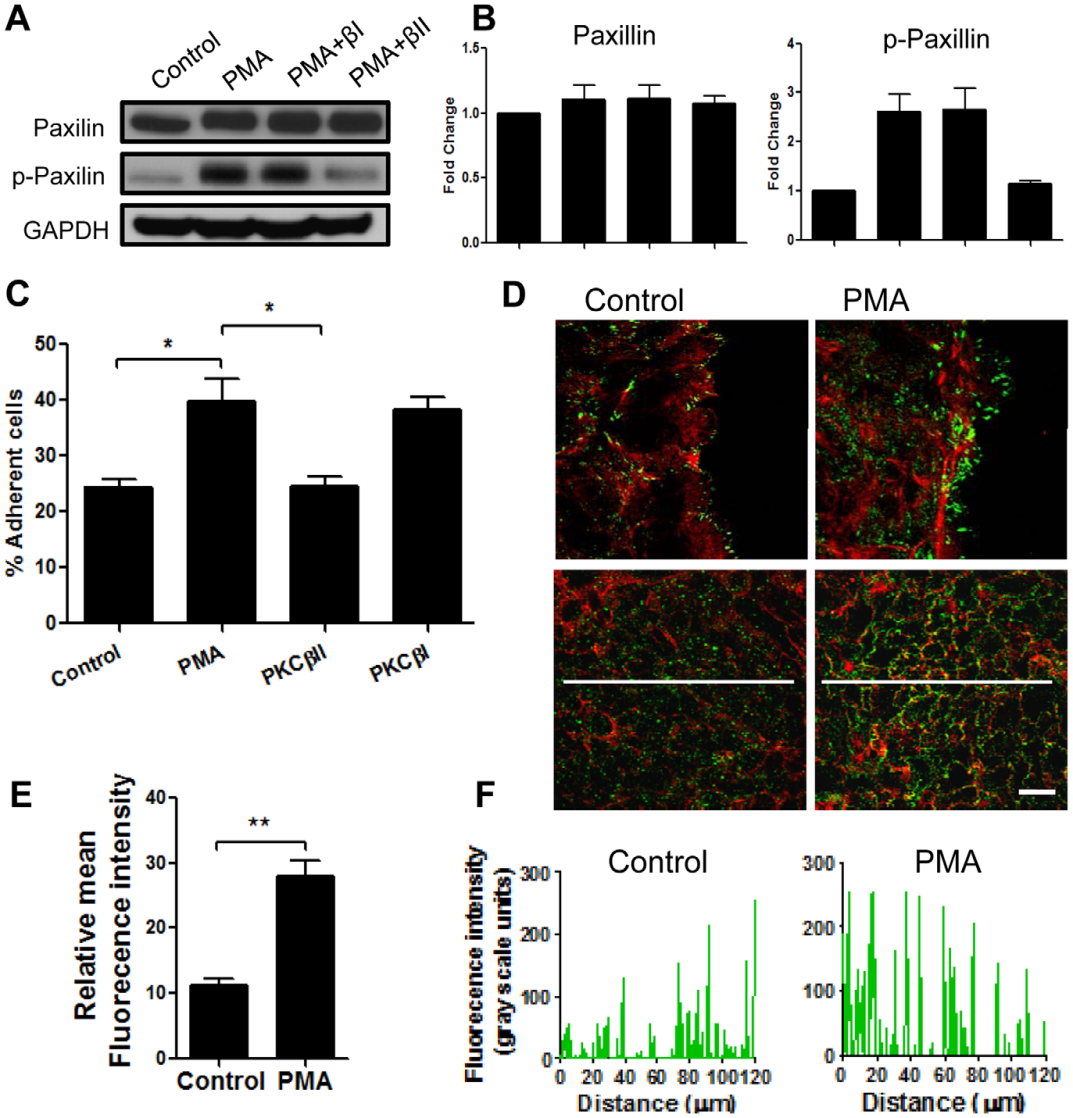
PKCβII activation by PMA increased assembly of focal adhesions. Scratch wounded IEC monolayers were allowed to migrate for 4 hours in the absence (control) or presence of PMA (PMA) and PKC isoforms inhibitors. The levels of total and phospo-paxillin were determined by immunoblotting (**A**) and quantified using densitometric analysis (**B**). The data are expressed as fold change relative to control after normalization to loading control. PMA treatment increased paxillin phosphorylation, which was attenuated by the inhibition of PKCβII (PMA+βII), but not PKCβI (PMA+βI). (**C**) Cell adhesion assay was used to examine IEC-matrix adhesion after PMA treatment in the presence or absence of specific inhibitors of PKC isoforms. PMA treatment significantly increased cell-matrix adhesion.This effect was reversed with inhibition of PKCβII, but not PKCβI. *significantly different (p<0.05). (**D**) To examine formation of focal adhesions by the cells at the leading edge (upper panels), and cells away from the wound (bottom panels) IECs were fixed and stained for F-actin (red) and phospho-paxillin (green). Representative images show increased phosphorylation of paxillin both at the lamellipodium and areas remote from the wound after PKC activation with PMA. The bar is 20 µm. (**E–F**) Quantification of the changes in the relative fluorescence intensity, as an indication of the changes in paxillin phosphorylation. (**E**) Average relative fluorescence intensity of 5 randomly placed lines (as shown in (D), bottom panels) per field of view, at least 7 fields per condition. N = 3 independent experiments. **significantly different (p<0.01). (**F**) Fluorescence intensity profiles of phospho-paxillin along representative white lines projected across fields of view (as shown on the images in (D), bottom panels) that were used for quantification in (E).

**Figure 7:**
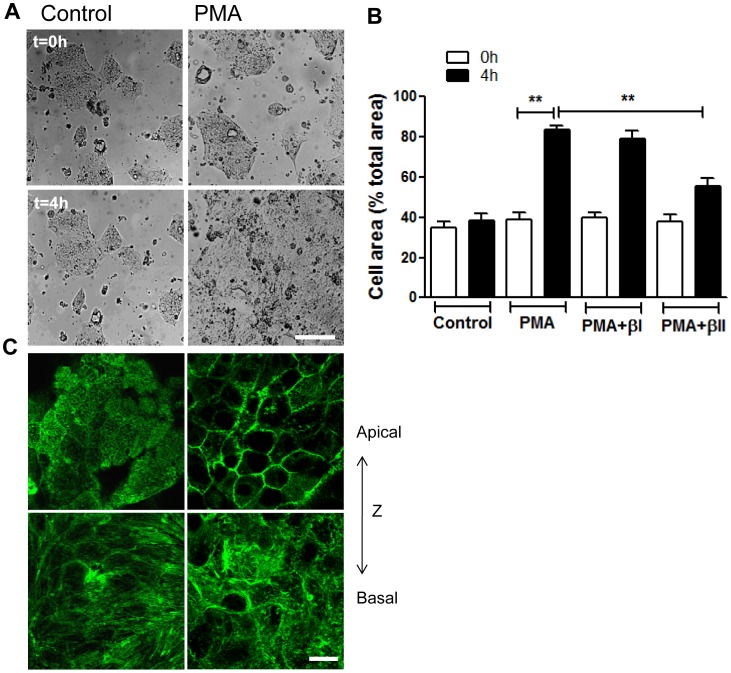
PKCβII mediates PMA induced epithelial cell spreading. (**A,B**) Subconfluent IECs monolayers (∼40% confluency) were allowed to spread on 13-mm collagen-coated coverslips in the absence (left panels) or presence (right panels) of PMA activation (200nM, 4 h), with the addition of PKCβII or PKCβI inhibitors as indicated. Cell area per field of view was measured before (t = 0 h, upper panels) and after treatment (t = 4 h, bottom panels). **(A)** Representative images demonstrating enhanced IEC spreading after PMA treatment. The bar is 50 µm. (**B**) Quantification of IEC spreading in the presence or absence of PKCβII (PMA+βII) or PKCβI (PMA+βI) inhibitors. Data presented as percent cell area/total area of the field of view. PMA induced cell spreading was significantly attenuated after inhibition of PKCβII. **significantly different (p<0.01). (**C**) Spreading IECs stimulated with PMA were fixed and stained for F-actin at t = 0 (left panels) and t = 4 h (right panels). Representative confocal microscopy images show most apical (upper panels) and most basal (bottom panels) distribution of F-actin. A dramatic reorganization of F-actin including loss of perijunctional actomyosin belt at the apical side, and redistribution of actin towards cell borders, as well as loss of stress fibers at the cell basal side in response to PMA treatment was observed. The bar is 20 µm.

Cellular protrusions at the leading edge have to adhere to the matrix to mediate forward cell movement. Such matrix adhesions in migrating cells are referred to as focal complexes. Paxillin, is a key protein that regulates focal cell matrix adhesion dynamics [9]. Therefore we asked whether PMA stimulation of IECs results in increased activation of paxillin (increased phosphorylation, Y118), and if this event is mediated by PKCβII. Using western blot analysis we found increased phospho-paxillin (∼2.6-fod) 4 hours after PMA stimulation (Fig. 6A,B). A small non-significant increase in total paxillin level (∼1.1-fold) was observed. Inhibition of PKCβII, but not PKCβI prevented the PMA induced increase in paxillin phosphorylation consistent with the effect of PMA and PKCβII inhibition on lamellipodia formation and wound closure. Similar results were observed with another key protein that is activated in focal complexes during cell adhesion and migration, focal adhesion kinse (FAK) [46]. PMA treatment significantly increased FAK phosphorylation (Y397), which was reversed with inhibition of PKCβII, but not PKCβI (Figure S4). As both phospho-paxillin and phospho-FAK were previously reported in engaged cell-matrix adhesions [46], we next investigated the role for PMA and PKCβII in IEC adhesion to extracellular matrix. PMA treatment increased cell-matrix adhesion (∼1.8-fold). This increase was reversed in the presence of PKCβII, but not PKCβI pharmacologic inhibitors (Fig. 6C), confirming that PKCβII activation by PMA enhances assembly of focal adhesions. Furthermore, as shown in representative immunofluorescence images (Fig. 6D upper panels) PMA induced lamellipodia had increased phospho-paxillin (Y118) that was observed in aggregates suggestive of focal adhesion plaques at the leading edge of spreading IECs. Interestingly, PMA treatment also significantly increased phosphorylation of paxillin in IECs located greater than 100 µm away from the wound (Fig. 6D bottom panels, and quantification Fig. 7E,F). Epithelial cells migrate as a sheet, where cells removed from the leadings edge also contribute to wound closure by cell spreading. As paxillin and FAK play an important role in cell spreading, our findings suggest that PMA-induced activation of paxillin in IECs is accompanied by spreading of the entire epithelial sheet and not just cells at the leading edge.

### PKCβII Mediates PMA Induced Epithelial Cell Spreading

We therefore next examined if PMA activation facilitates intestinal epithelial cell spreading, and whether this is a PKCβII dependent process. To assay for cell spreading, ∼40% confluent T84 IECs were stimulated with PMA for 4 hours in the presence or absence of PKCβII inhibitor. The surface area of the migrating sheet before and after treatment was measured and compared to non-activated cells. We found no significant change in cell area of control monolayers after 4 hours, however PMA activation resulted in dramatic increase in cell area (from 39.2±3.2% to 83.5±2.2%, Fig. 7B and representative images, Fig. 7A). PMA induced cell spreading was accompanied by F-actin cytoskeletal reorganization that included, loss of the apical brush border actin and redistribution of F-actin towards cell borders. This was also accompanied by loss of basal stress fibers (Fig. 7C). Importantly, PMA induced cell spreading (Fig. 7A,B), as well as F-actin rearrangement (not shown) was significantly attenuated after inhibition of PKCβII but not PKCβI or PKCγ. Interestingly, PKCβII inhibition was not sufficient to fully reverse the PMA-induced increase in cell area, suggesting that a minor component of this increase may be due to increased proliferation after PMA treatment (as shown in Fig. 4A,B). Indeed, we confirmed that PKCβII inhibition had minimal effects on IEC proliferation (data not shown). These findings suggest that PMA induced enhanced wound closure was primarily due to increased cell spreading and migration, and that PKCβII activation in response to PMA treatment enhances epithelial wound closure through increased cell spreading.

**Figure 8:**
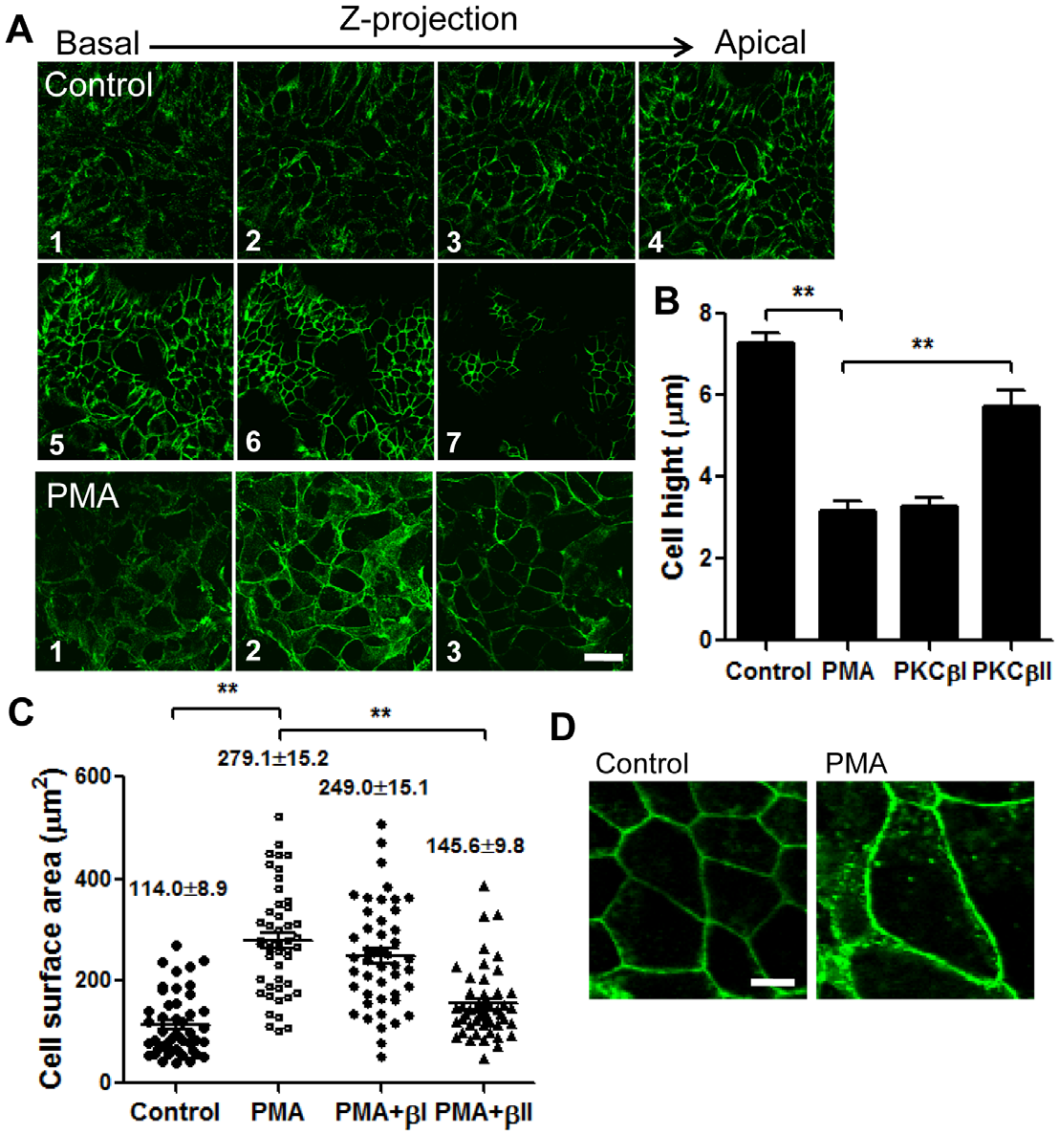
Spreading IECs were fixed and stained for E-Cadherin before (control) and after PMA activation (PMA) in the presence or absence of PKC blockers as indicated. (A) Representative confocal images acquired in series in Z-direction show changes in cell height before (control, 1–7) and after PMA treatment (PMA, 1–3). The bar is 20 µm. (B) Quantification of cell height in 7 randomly selected fields per condition, N = 3 independent experiments. **significantly different (p<0.01). (C) The changes in the surface area of individual cells under the specified conditions were quantified from images obtained using confocal microscopy, and positioning the focal plane at the apical IEC surface, and depicted in the representative images (D). The bar is 10 µm. The height of IECs was significantly decreased, and the approximate cell surface area was significantly increased after PKC activation by PMA consistent with cell spreading. In both cases inhibition of PKCβII significantly attenuated these changes. **significantly different (p<0.01).

We further confirmed the effect of PKC activation on cell spreading by detailed examination of cell morphology in control and PMA activated subconfluent and spreading T84 cells. Confocal microscopy and serial image acquisition in X-Z plane of IECs immunostained for the adherens junction protein, E-cadherin revealed that control cell height was 7.2±0.3 µm, while cells exposed to PMA were significantly flattened (cell height, 3.2±0.3 µm, Fig. 8B and Fig. 8A, 1–7 and Fig. 8A, 1–3, respectively). Additionally, the surface area of individual spreading cells was significantly increased after PMA treatment (from 114.0±8.9 to 279.1±15.2 µm^2^, Fig. 8C, and representative images Fig. 8D) indicative of cell spreading. In agreement with cell spreading assay results (Fig. 7A,B), inhibition of PKCβII attenuated both the decrease in cell height and the increase in cell surface area, confirming the role for PKCβII in epithelial cell spreading.

## Discussion

Epithelial cells lining the intestine protect the underlying tissue compartment from luminal contents including foreign antigens and bacteria. Rapid repair of epithelial injury, as seen secondary to inflammation and ischemia is essential for maintaining barrier function and tissue homeostasis. IEC wound repair involves multiple cellular signaling events including PKC activation.

In the current work we investigated the effect of PKC activation on IEC wound healing after exogenous stimulation with PMA. PKC activation has been shown to enhance proliferation of various tumor cell lines, and is often associated with tumorigenesis [20], [21]. However, we observed that in IECs, PMA activation of PKC family members enhanced wound closure independently of cell proliferation. The effect of PMA on IEC wound closure was due to enhanced cell spreading and migration, and was specifically mediated by PKCβII. PMA treatment of migrating IECs resulted in increased lamellipodial extrusions at the leading edge. We also observed increased paxillin phosphorylation mediated by PKCβII suggestive of increased focal adhesion dynamics in migrating cells. PKCβII inhibition using pharmacological inhibitors significantly attenuated both PMA-induced cell spreading/formation of lamellipodia, and paxillin phosphorylation, consequently decreasing PMA induced wound closure.

It has been previously recognized that the severity and size of an injury will determine the mechanisms involved in the healing process. For example, superficial small injury heals by spreading and migration of the epithelium [47], while larger wounds, as observed in disorders such ulcerative colitis and Crohn’s disease require an additional component of cell proliferation [2]. We observed that PMA effect on IEC wound closure was most dramatic within the first 24 hours (Fig. 1), consistent with increased cell spreading and migration rather than increased cell proliferation. Thus our in-vitro model of epithelial wound repair simulates the repair mechanism characteristic of small to medium sized wounds in the intestinal tract.

IEC migration requires reorientation of the leading edge cells in the direction of the wound through reorientation of microtubule organizing center (MTOC) and Golgi apparatus [6], as well as formation of membrane protrusions (lamellipodium) [8]. This is initiated by localized activation of Rho-family small GTPases, including Cdc42 and Rac [6]. PKC isoforms have been previously implicated in activation of Rho family small GTPases [48], [49], thus we asked whether PMA induced increase in IEC wound closure was due to enhanced directional polarity and increased lamellipodia formation, and whether inhibition of PKCβII attenuated the PMA induced wound closure via inhibition of either or both of these processes. While we found no significant difference in directional polarity of migrating cells (orientation of golgi apparatus, Fig. 5A,B), PMA treatment significantly enhanced formation of lamellipodia (Fig. 5C,D). Importantly, this was mediated through activation of PKCβII, as its inhibition abolished this effect. It has been previously shown that the directional movement of fibroblasts involves Cdc42, while formation of membrane protrusions is mediated by Rac1 [50]. These findings suggest that in IECs PMA activation of PKCβII might influence activation of Rac1 but not Cdc42. In support of this, PMA activation of Rac1 but not Cdc42 has been previously documented in other epithelial cell lines such as Cos1 and A431 cells [51].

Extended lamellipodia serve as footholds for forward propulsion of cell, thus they are intimately linked to assembly of focal adhesion complexes [8]. Indeed we found dramatic increase in paxillin and FAK phosphorylation, which was decreased by PKCβII inhibition (Fig. 6A,B).

Interestingly, we also observed a significant increase in phosphorylation of paxillin in regions adjoining the leading edge cells (greater than 100 µm away, Fig. 6D bottom panels, and quantification Fig. 6E,F) following PMA treatment. Increased paxillin phosphorylation has been previously associated with enhanced cell adhesion and spreading [52], [53]. The pattern of paxillin phosphorylation in the IEC monolayers suggests that PMA promotes spreading and migration of the epithelium as a sheet, and does not affect exclusively the leading edge cells. This is consistent with earlier observations that IECs migrate as a cohesive sheet, and not as individual cells. Indeed, PMA induced a dramatic increase in T84 IECs spreading (Figs. 7 and 8), which was blocked by inhibition of PKCβII (Fig. 7). We show that after PMA activation the spreading cells were significantly flattened and had increased surface area (Fig. 8). Thus, individual spreading cells in the epithelial sheet covered larger, more than twice the area compared to tall columnar stationary polarized cells.

In summary we highlight the involvement of PKCs, specifically PKCβII in rapid healing of IECs in response to endogenous stimulation, through enhanced cell spreading. These findings outline the importance of epithelial cell spreading in migrating sheet during wound closure and intestinal barrier recovery.

## Supporting Information

Figure S1 **PKC activation with PMA enhances epithelial wound healing.** Confluent Caco-2 IEC monolayers were wounded by introduction of a single linear scratch wound. Wound closure was measured over 48 h in unstimulated (control) and PMA activated monolayer as detailed in the methods section. Similarly to T84 monlayers, PMA treatment induced a dramatic increase in wound closure. N = 4 independent experiments. **significantly different (p<0.01).
(TIF)

Figure S2 **PKCβII mediates PMA induced increase in wound closure.** Confluent IEC monolayers were wounded by introduction of a single linear scratch wound, preincubated with specified PKC-isoform inhibitors (KIBI31-1, PKCβI inhibitor (1 µM, PKCβI); KIBII31-1 (5 µM, PKCβII); KIG31-1, PKCγ inhibitor (5 µM, PKCβγ) for 1 hour and stimulated with PMA (200 nM). The area of the wound was measured at 12 and 24 hours. Inhibition of PKCβII, but not inhibition of other members of the classical PKCs significantly diminished PMA enhanced wound closure. *significantly different from control (PMA alone, p<0.05). **significantly different from control (PMA alone, p<0.01). N = 3 independent experiments.
(TIF)

Figure S3 **PKCβII expression and distribution** **mediates** **PMA induced increase in wound closure. (A)** To assess the effect of PMA treatment on PKCβII expression confluent IEC monolayers were wounded by introduction of multiple linear scratch wounds (as described in methods), treated with PMA (200 nM), and were prepared for western blot analysis at the indicated time points. Immunoblots are representative of 3 independent experiments. Expression of PKCβII was significantly reduced16 hours after PMA treatment. **(B)** Confluent IEC monolayers were wounded by introduction of a single linear scratch wound. Wound closure after PMA treatment was measured at the indicated time points. The data presented as fold change above control (immediately after wounding). PMA-dependent effect on wound repair was significantly decreased at 16 hours and 24 hours compared to 12 hours treatment. *significantly different (p<0.05). **significantly different (p<0.01). N = 3 independent experiments. **(C)** PKCβII translocation to cell membrane in leading edge cells after PMA treatment (200 nM, 4 hr), but not in control wounds was confirmed by immunofluorescence labeling and confocal microscopy. The images are representative of 3 independent experiments. The bar is 20 µm.
(TIF)

Figure S4 **PKCβII activation by PMA increased assembly of focal adhesions.** Scratch wounded IEC monolayers were allowed to migrate for 4 hours in the absence (control) or presence of PMA (PMA) and PKC isoforms inhibitors. The levels of total and phospo-FAK were determined by immunoblotting **(A)** and quantified using densitometric analysis **(B)**. The data are expressed as fold change relative to control after normalization to loading control. PMA treatment increased FAK phosphorylation, which was attenuated by the inhibition of PKCβII (PMA+βII), but not PKCβI (PMA+βI).
(TIF)
