# Supplementary figures and images for: Activation of PKCβII by PMA Facilitates Enhanced Epithelial Wound Repair through Increased Cell Spreading and Migration
Source: PLoS One. 2013 Feb 11;8(2):e55775. doi: 10.1371/journal.pone.0055775 (PMC3569445; doi:10.1371/journal.pone.0055775)

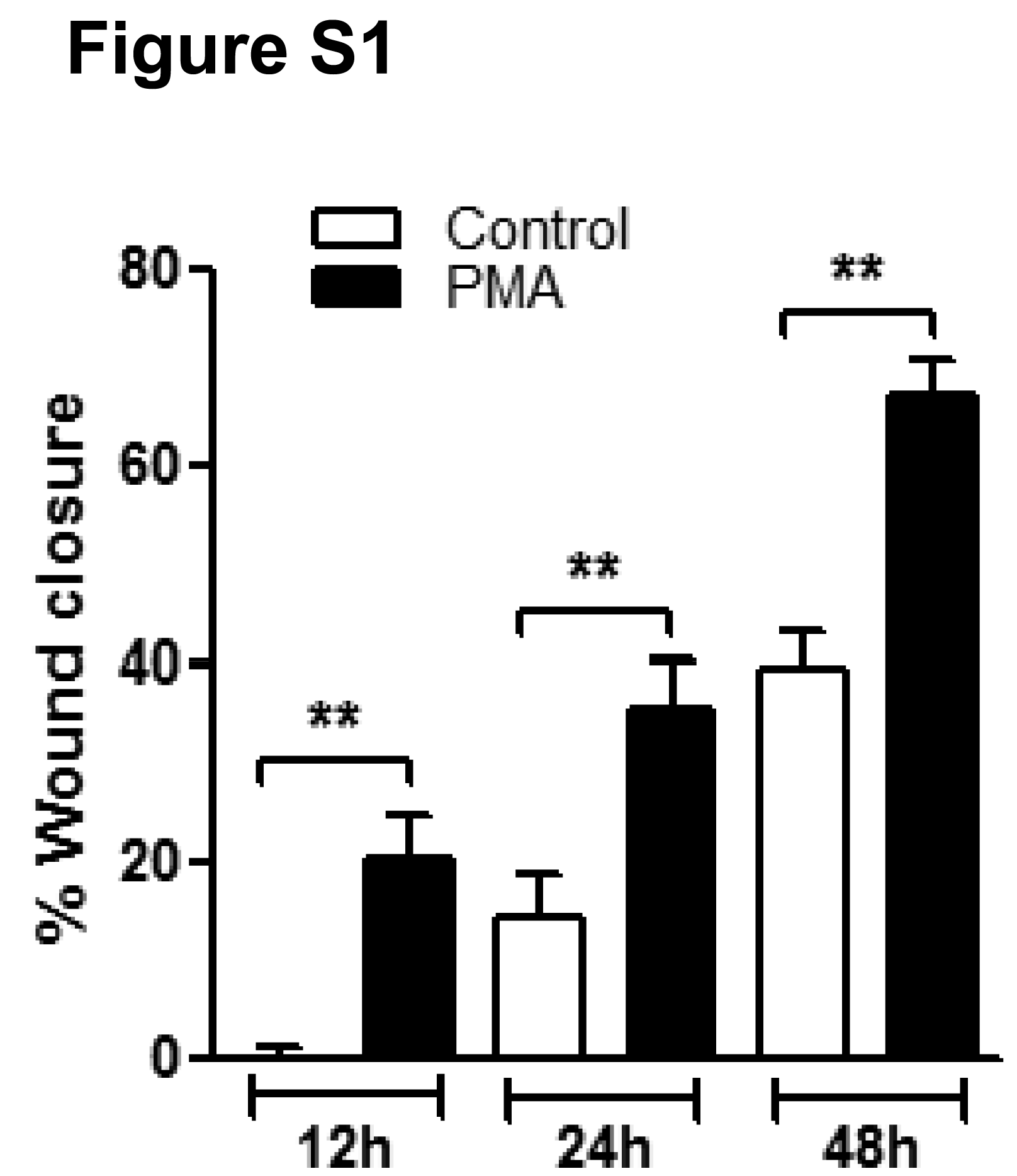

Supplement: Figure S1 — PKC activation with PMA enhances epithelial wound healing. Confluent Caco-2 IEC monolayers were wounded by introduction of a single linear scratch wound. Wound closure was measured over 48 h in unstimulated (control) and PMA activated monolayer as detailed in the methods section. Similarly to T84 monlayers, PMA treatment induced a dramatic increase in wound closure. N = 4 independent experiments. **significantly different (p<0.01). (TIF) [file pone.0055775.s001.tif]

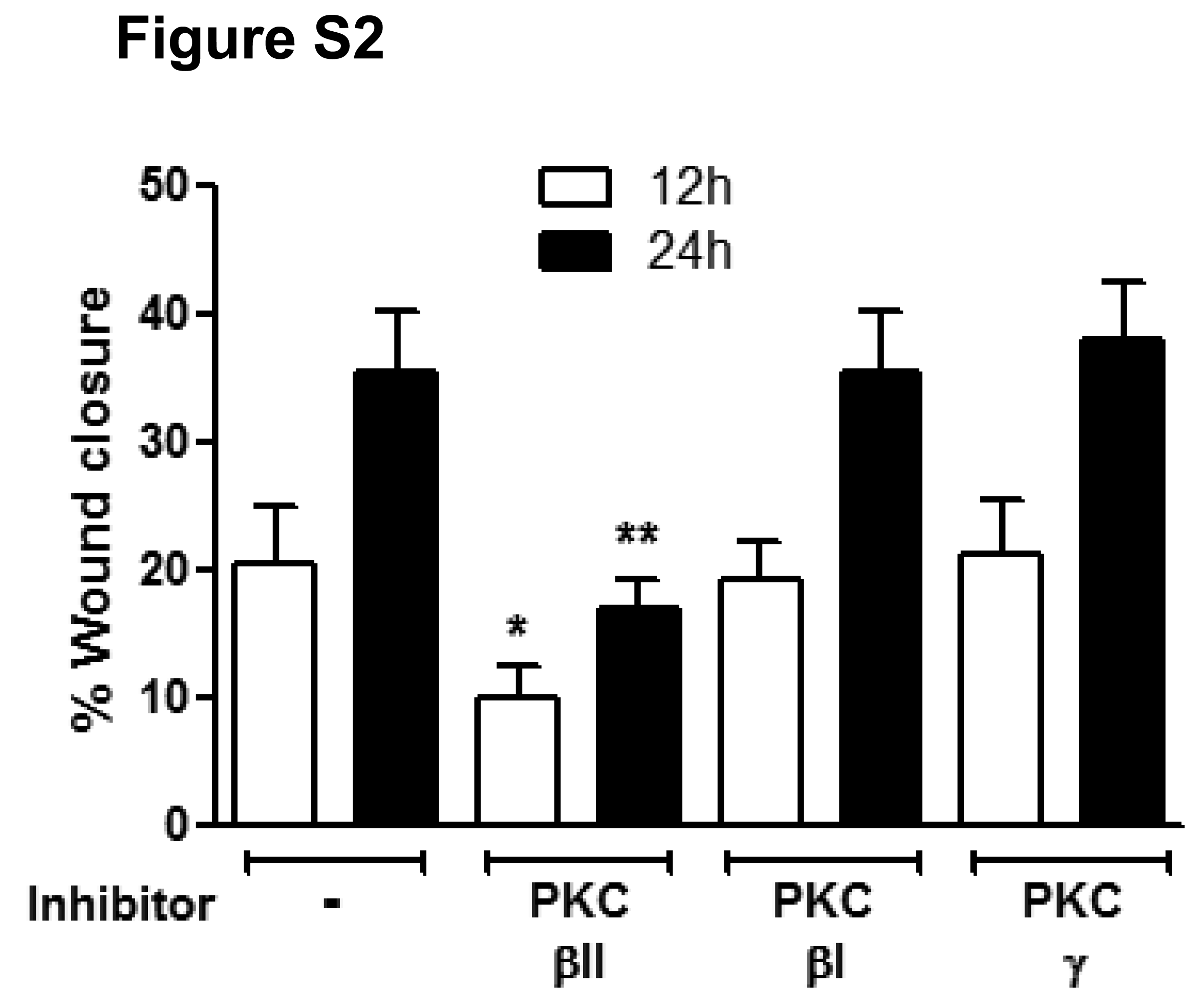

Supplement: Figure S2 — PKCβII mediates PMA induced increase in wound closure. Confluent IEC monolayers were wounded by introduction of a single linear scratch wound, preincubated with specified PKC-isoform inhibitors (KIBI31-1, PKCβI inhibitor (1 µM, PKCβI); KIBII31-1 (5 µM, PKCβII); KIG31-1, PKCγ inhibitor (5 µM, PKCβγ) for 1 hour and stimulated with PMA (200 nM). The area of the wound was measured at 12 and 24 hours. Inhibition of PKCβII, but not inhibition of other members of the classical PKCs significantly diminished PMA enhanced wound closure. *significantly different from control (PMA alone, p<0.05). **significantly different from control (PMA alone, p<0.01). N = 3 independent experiments. (TIF) [file pone.0055775.s002.tif]

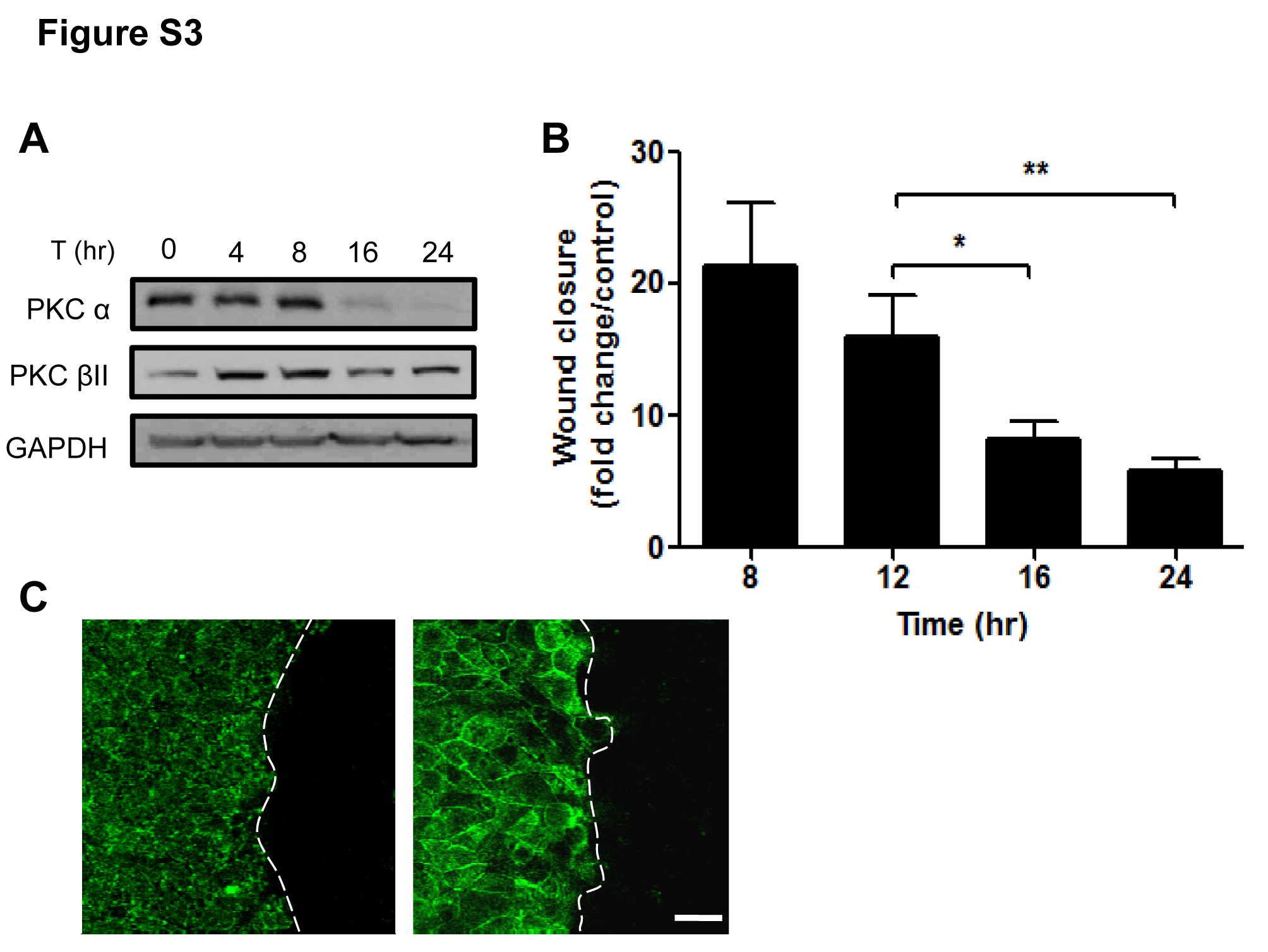

Supplement: Figure S3 — PKCβII expression and distribution mediates PMA induced increase in wound closure. (A) To assess the effect of PMA treatment on PKCβII expression confluent IEC monolayers were wounded by introduction of multiple linear scratch wounds (as described in methods), treated with PMA (200 nM), and were prepared for western blot analysis at the indicated time points. Immunoblots are representative of 3 independent experiments. Expression of PKCβII was significantly reduced16 hours after PMA treatment. (B) Confluent IEC monolayers were wounded by introduction of a single linear scratch wound. Wound closure after PMA treatment was measured at the indicated time points. The data presented as fold change above control (immediately after wounding). PMA-dependent effect on wound repair was significantly decreased at 16 hours and 24 hours compared to 12 hours treatment. *significantly different (p<0.05). **significantly different (p<0.01). N = 3 independent experiments. (C) PKCβII translocation to cell membrane in leading edge cells after PMA treatment (200 nM, 4 hr), but not in control wounds was confirmed by immunofluorescence labeling and confocal microscopy. The images are representative of 3 independent experiments. The bar is 20 µm. (TIF) [file pone.0055775.s003.tif]

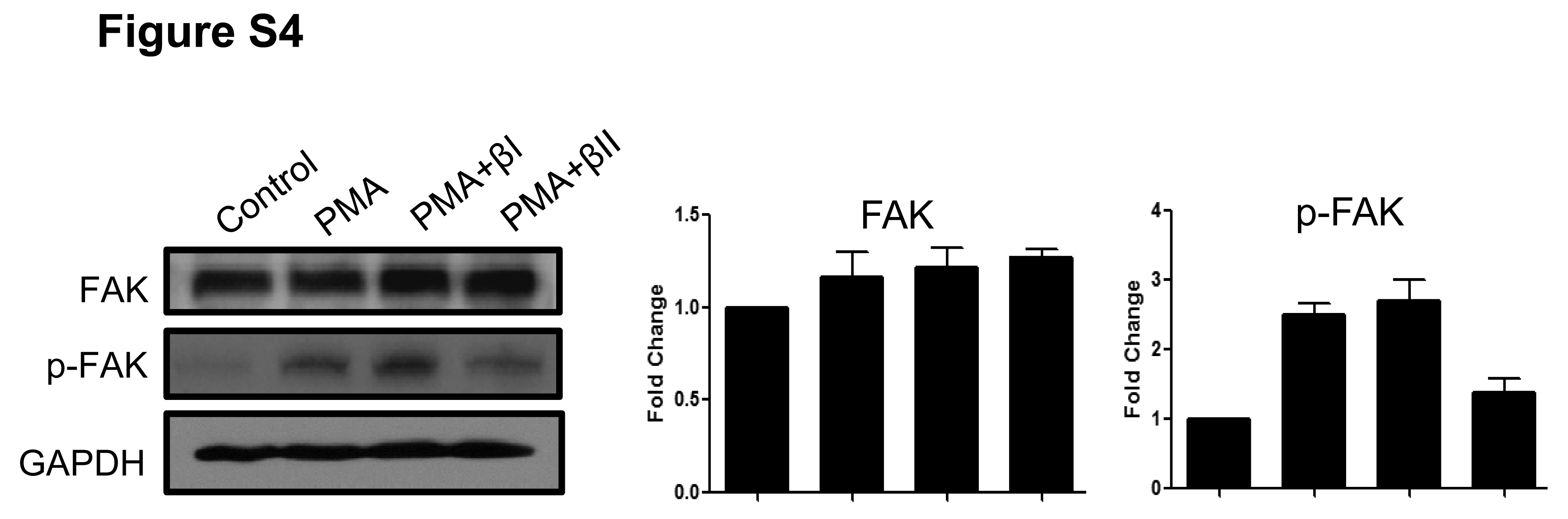

Supplement: Figure S4 — PKCβII activation by PMA increased assembly of focal adhesions. Scratch wounded IEC monolayers were allowed to migrate for 4 hours in the absence (control) or presence of PMA (PMA) and PKC isoforms inhibitors. The levels of total and phospo-FAK were determined by immunoblotting (A) and quantified using densitometric analysis (B). The data are expressed as fold change relative to control after normalization to loading control. PMA treatment increased FAK phosphorylation, which was attenuated by the inhibition of PKCβII (PMA+βII), but not PKCβI (PMA+βI). (TIF) [file pone.0055775.s004.tif]
